# Creativity and psychopathology: are there similar mental processes involved in creativity and in psychosis-proneness?

**DOI:** 10.3389/fpsyg.2014.01211

**Published:** 2014-10-24

**Authors:** Andreas Fink, Mathias Benedek, Human-F. Unterrainer, Ilona Papousek, Elisabeth M. Weiss

**Affiliations:** ^1^Institute of Psychology, University of GrazGraz, Austria; ^2^Center of Integrative Addiction Research (Grüner Kreis Society)Vienna, Austria

**Keywords:** creativity, psychopathology, cognitive control, schizotypy, originality

The putative link between creativity and psychopathology is nearly as legendary and mysterious as the long-standing view from antique mythology according to which eminent creative achievements were perceived as the result of a “higher power,” mostly caused by inspiration by muses. In fact, there are many illustrative examples of creative people who suffer(ed) from serious mental disorders, leading some authors to the notion that “… madness may be the price for possessing one of the most sublime human gifts” (Barrantes-Vidal, 2004, p. 59). Within the scientific domain, literature reviews came to quite different conclusions, assuming no, only a weak or even a strong association between creativity and psychopathology (Barrantes-Vidal, 2014, p. 170), ranking this topic into the most controversially discussed issues in the field of creativity. Nevertheless, there appears to be some consensus that at least less severe manifestations of psychopathology are associated with creativity (e.g., Barrantes-Vidal, 2004; Claridge and Blakey, 2009; Nelson and Rawlings, 2010). As we will briefly indicate in the following, especially people who are prone to psychosis (characterized in its most severe manifestation by e.g., delusions, hallucinations, disorganized thought, negative symptoms; see e.g., Heckers et al., 2013, for a review of the domains of psychopathology that define psychosis) have been found to show elevated levels of creativity.

## Creativity and psychosis-proneness

According to Carson (2011) empirical evidence for an association between creativity and psychopathology can be found from the latter half of the last century onward, stimulated by two separate studies. Heston (1966) investigated the psycho-social adjustment of children of mothers with schizophrenia relative to matched control subjects and he reported that the former were “notably successful adults” (p. 819), as they possessed artistic talents and showed imaginative adaptations to life. Some years later, Karlsson (1970) reported that relatives of psychotic patients (schizophrenics, manic depressives) had a higher rate of listing in Who is Who, both based on a general listing and on creative endeavors. Since then, researchers began to examine the incidence of psychopathology in highly successful, creative achievers. As one of the landmark studies in this field, Andreasen (1987) for instance found a higher rate of mood disorders (involving bipolar disorder) in prominent writers as compared to a matched control group. Most interestingly, there was also a higher prevalence of mood disorders and creativity in the first-degree relatives of the writers as compared to the relatives of the control subjects, suggesting that the “mad genius” trait might be genetically heritable (Andreasen, 1987). Recent epidemiological studies with large sample sizes confirm the association between professional authors and psychiatric disorders, especially schizophrenia and bipolar disorder, and indicate a familial association between overall creative professions for schizophrenia, bipolar disorder, anorexia nervosa, and possibly autism (Kyaga et al., 2011, 2013).

The idea that at least some facets of psychopathology could be associated with creativity has also received some support from the psychometric research tradition. Eysenck's (1995) psychoticism (P) dimension for instance, a personality trait involving cold, un-empathic, aggressive, and impulsive behavior, has been observed as being substantially associated with various creativity-related demands, particularly with the originality facet of creativity (Abraham et al., 2005; Fink et al., 2012; for review see Acar and Runco, 2012). Eysenck's P dimension is thought to underlie a variety of psychotic disorders (Eysenck, 1995; but see also Chapman et al., 1994) and it “… differs from psychosis by not being pathological and hence enabling people to use remote associations in a constructive way (Eysenck, 1995, p. 244). While individuals scoring low on P are characterized by e.g., conformity or conventionality, high P scorers show traits such as impulsivity, aggression or hostility, and therefore a high tendency toward unconformity, which could possibly provide some explanation for the observed relationship between originality and psychoticism.

Creativity has also been investigated in relation to schizotypy, which involves traits such as unusual experiences, cognitive disorganization, introvertive anhedonia (lack of enjoyment/interpersonal domain) or impulsive non-conformity (Claridge and Blakey, 2009), and is known as increased vulnerability of developing psychotic disorders (e.g., Claridge, 1997; Fisher et al., 2004; Nettle, 2006). Studies yielded evidence that some facets of schizotypy (positive symptoms such as unusual, hallucinatory experiences) may be linked to psychometrically determined creativity (e.g., Claridge and Blakey, 2009). Similarly, the studies of Nettle (2006) and Nelson and Rawlings (2010) found elevated levels of positive schizotypy in a sample of artists. In light of such findings, it has been argued that some cognitive styles may be similar between creative and psychotic thinking (Keefe and Magaro, 1980; Eysenck, 1995; Carson, 2011). Such common cognitive processes can be assumed in “overinclusiveness” of thinking (Eysenck, 1995) or reduced latent inhibition which might both enable that more stimuli (also such that are not directly task-relevant) enter conscious awareness and may thus people allow to “… perceive and describe what remains hidden from the view of others” (Carson et al., 2003, p. 499; for a detailed discussion on these processes see Eysenck, 1995; Carson, 2011). In using functional magnetic resonance imaging, Fink et al. (2014) showed that originality and schizotypy were associated with similar functional brain activity patterns during creative ideation, which also adds some evidence to the idea that similar mental processes may be implicated in creativity as well as in psychosis-proneness. Quite similarly, Jung et al. (2010) investigated white matter integrity (assessed by Fractional Anisotropy, FA) in a sample of young healthy volunteers and they found that lower levels of FA within left inferior white matter (especially the anterior thalamic radiation) were associated with higher divergent thinking performance. Jung et al. (2010) refer to studies involving schizophrenic and bipolar patients which likewise found reduced FA in similar brain regions (Sussmann et al., 2009), demonstrating potential overlap between the neural substrates of both creative cognition and psychopathology or psychosis.

## Creativity and adaptive traits

It seems that some mental processes might be quite similar between creative and psychotic thinking, but current literature does not allow for strong conclusions, not least due to severe methodological and conceptual challenges in this field (Schlesinger, 2009; Dietrich, 2014; Simonton, 2014). Importantly, research from the psychological research tradition also provides evidence that creativity is amongst others closely associated with intelligence (Jauk et al., 2013), domain-specific knowledge/expertise (e.g., Weisberg, 1999), motivation (Collins and Amabile, 1999), and thus with highly adaptive traits (see also Simonton, 2000). In addition, the burgeoning field of neuroscience studies on creativity reveals that this ability is associated with “ordinary” (rather than psychopathological) brain processes that are likewise seen in various cognitive ability domains (e.g., Fink and Benedek, 2014). And finally, creativity involves various “positive” personality traits such as openness, broad interests or self-confidence (Barron and Harrington, 1981; Feist, 1998).

A particular conceptual challenge in this field is that any association of creative cognition/divergent thinking with psychosis-proneness often implicates disorganization of thought and impaired cognitive control, which may facilitate the loosening of constraints and conventional ways of thinking, and thus the generation of more distant, unusual or novel associations. At first sight, however, this appears to be at odds with a large amount of empirical evidence on a positive relationship between creativity (in terms of divergent thinking ability) and intelligence (Kim, 2005; Nusbaum and Silvia, 2011; Jauk et al., 2013, 2014), and highly effective executive functioning (e.g., working memory and cognitive inhibition; Benedek et al., 2012, 2014), rather indicating a crucial role of cognitive control in creative thought.

## Creativity—a controlled explosion of mind

So, on the basis of the reviewed studies, what are we to conclude about the putative link between creativity and psychopathology? Carson (2011) assumes that high levels of intelligence and working memory capacity act as “protective factors” in the sense that they facilitate more efficient processing of available information produced by “vulnerability factors” such as novelty seeking or reduced latent inhibition. Similar to that idea, both higher and lower levels of cognitive control may be implicated in creativity, but at different stages of the creative process (cf. Kris' supposition of primary vs. secondary process cognition in creative individuals; Kris, 1952). The disposition for the generation of unusual representations may be particularly conducive to creative thought, if these representations can be organized and elaborated effectively. This point can be further illustrated by invoking the Geneplore model (Finke et al., 1992), which distinguishes between generation and exploration phases during creative idea generation, where the latter phase is concerned with the exploration, elaboration, and evaluation of initially generated mental representations. Within this framework, some psychopathological traits may generally be thought to feed the generation stage, while at the exploration stage high cognitive control is needed to separate the wheat from the chaff, and to elaborate relevant unusual representations toward actually creative ideas (cf. Kaufman and Paul, 2014).

Merten and Fischer (1999) provide interesting evidence in favor of this assumption. They compared the association behavior of creative people (professional writers and actors) to individuals suffering from schizophrenia and normal controls. They found that, given the instruction to be original, the creative group showed highly original response behavior, similar or even more original than that of individuals with schizophrenia. However, when instructed to generate common associations, the creative group performed similar to the control group, while the schizophrenic group still showed higher unusualness. Finally, the creative group was also better able to assess the commonness of their responses than individuals with schizophrenia. These findings demonstrate that creative people show a similar disposition for the generation of novelty like individuals suffering from schizophrenia, but they also show better control of their ideational output, including the evaluation of appropriateness of their responses.

The Merten and Fischer (1999) study also points to a potentially important methodological issue in the psychometric study of creativity and psychopathology. According to common definitions (Runco and Jaeger, 2012), novelty is a central ingredient of creativity, because common ideas can never be considered as creative. However, the second necessary criterion is the appropriateness or the efficacy of an idea, which in turn determines whether an idea or a product is actually creative or just absurd. It may thus well be the case that studies using divergent thinking tasks (i.e., common indicators of creative cognitive potential) will likely fail to observe the complete picture of differences when simply scoring for ideational fluency or uniqueness, as these scores disregard the creative quality of ideas. Unfortunately, such coarse scorings of divergent thinking tasks are still quite common, sometimes justified by an apparent lack of discriminant validity of the scores derived from subjective scoring methods. However, methods for the efficient scoring of the creative quality of ideas independent of the confounding influence of fluency are readily available (Silvia et al., 2008; Benedek et al., 2013).

We hence assume that available evidence for a relationship of psychosis-proneness with creativity, particularly within the psychometric research tradition, may sometimes be restricted to unusualness. But any trait supporting the generation of unusual representations may be highly conducive for the creativity of thought, if it concurs with the necessary cognitive control to guide evaluation and elaboration at the exploration stage of creative idea generation (see also Carson, 2011; Kaufman and Paul, 2014). We would thus more likely succeed in our understanding of the putative link between creativity and psychopathology if we base our conclusions more strongly on carefully designed empirical studies, which focus on specific cognitive and neural processes that may be similar or even shared between creative and psychotic thinking. This would require the application of well-proven methods and paradigms in carefully selected samples of both clinical and non-clinical samples of participants. In this context, researchers also need to carefully distinguish between different creativity domains (e.g., artistic vs. scientific), given that creative people in different domains show different personality profiles (Feist, 1998), and given the affinity of psychosis-proneness to the artistic creativity domain (Nettle, 2006; Nelson and Rawlings, 2010; Kyaga et al., 2011, 2013). Taken together, such an approach could identify some of the complex cognitive and neural processes involved in both creativity and psychopathology, and would have the potential to draw a more concise picture of some mechanisms overlapping between both constructs, rather than linking creativity generally to “madness.”

### Conflict of interest statement

The authors declare that the research was conducted in the absence of any commercial or financial relationships that could be construed as a potential conflict of interest.

## References

[B1] Abraham A. Windmann S. Daum I. Güntürkün O. (2005). Conceptual expansion and creativity imagery as a function of psychoticism. Conscious. Cogn. 14, 520–534. 10.1016/j.concog.2004.12.003 15925521

[B2] Acar S. Runco M. A. (2012). Psychoticism and creativity: a meta-analytic review. Psychol. Aesthet. Crea. Arts 6, 341–350. 10.1037/a0027497

[B3] Andreasen N. C. (1987). Creativity and mental illness: prevalence rates in writers and their first-degree relatives. Am. J. Psychiatry 144, 1288–1292. 3499088 10.1176/ajp.144.10.1288

[B4] Barrantes-Vidal N. (2004). Creativity and madness revisited from current psychological perspectives. J. Conscious. Stud. 11, 58–78

[B5] Barrantes-Vidal N. (2014). Creativity and the spectrum of affective and schizophrenic psychoses, in Creativity and mental illness, ed Kaufman J. C. (Cambridge: Cambridge University Press), 169–204. 10.1017/CBO9781139128902.013

[B6] Barron F. Harrington D. M. (1981). Creativity, intelligence and personality. Annu. Rev. Psychol. 32, 439–476. 10.1146/annurev.ps.32.020181.002255

[B7] Benedek M. Franz F. Heene M. Neubauer A. C. (2012). Differential effects of cognitive inhibition and intelligence on creativity. Pers. Individ. Dif. 53, 480–485. 10.1016/j.paid.2012.04.014 22945970 PMC3387381

[B8] Benedek M. Jauk E. Sommer M. Arendasy M. Neubauer A. C. (2014). Intelligence, creativity, and cognitive control: the common and differential involvement of executive functions in intelligence and creativity. Intelligence 46, 73–83. 10.1016/j.intell.2014.05.007 25278640 PMC4175011

[B9] Benedek M. Mühlmann C. Jauk E. Neubauer A. C. (2013). Assessment of divergent thinking by means of the subjective top-scoring method: effects of the number of top-ideas and time-on-task on reliability and validity. Psychol. Aesthet. Crea. Arts 7, 341–349. 10.1037/a0033644 24790683 PMC4001084

[B10] Carson S. H. (2011). Creativity and psychopathology: a shared vulnerability model. Can. J. Psychiatry 56, 144–153. 21443821 10.1177/070674371105600304

[B11] Carson S. H. Peterson J. B. Higgins D. M. (2003). Decreased latent inhibition is associated with increased creative achievement in high-functioning individuals. J. Pers. Soc. Psychol. 85, 499–506. 10.1037/0022-3514.85.3.499 14498785

[B12] Chapman J. P. Chapman L. J. Kwapil T. R. (1994). Does the Eysenck psychoticism scale predict psychosis? A ten year longitudinal study. Pers. Individ. Dif. 17, 369–375. 10.1016/0191-8869(94)90284-4

[B13] Claridge G. (1997). Schizotypy: Implications for Illness and Health. Oxford, NY: Oxford University Press

[B14] Claridge G. Blakey S. (2009). Schizotypy and affective temperament: relationships with divergent thinking and creativity styles. Pers. Individ. Dif. 46, 820–826. 10.1016/j.paid.2009.01.015

[B15] Collins M. A. Amabile T. M. (1999). Motivation and creativity, in Handbook of Creativity, ed Sternberg R. J. (Cambridge, UK: Cambridge University Press), 297–312

[B16] Dietrich A. (2014). The mythconception of the mad genius. Front. Psychol. 5:79. 10.3389/fpsyg.2014.00079 24616710 PMC3935122

[B17] Eysenck H. J. (1995). Creativity as a product of intelligence and personality, in International Handbook of Personality and Intelligence, eds Saklofske D. H. Zeidner M. (New York; London: Plenum Press), 231–247. 10.1007/978-1-4757-5571-8_12

[B18] Feist G. J. (1998). A meta-analysis of personality in scientific and artistic creativity. Pers. Soc. Psychol. Rev. 2, 290–309. 10.1207/s15327957pspr0204_5 15647135

[B19] Fink A. Benedek M. (2014). EEG alpha power and creative ideation. Neurosci. Biobehav. Rev. 44, 111–123. 10.1016/j.neubiorev.2012.12.002 23246442 PMC4020761

[B20] Fink A. Slamar-Halbedl M. Unterrainer H. F. Weiss E. (2012). Creativity: genius, madness, or a combination of both? Psychol. Aesthet. Crea. Arts 6, 11–18. 10.1037/a0024874

[B21] Fink A. Weber B. Koschutnig K. Benedek M. Reishofer G. Ebner F.. (2014). Creativity and schizotypy from the neuroscience perspective. Cogn. Affect. Behav. Neurosci. 14, 378–387. 10.3758/s13415-013-0210-6 24022793

[B22] Finke R. A. Ward T. M. Smith S. M. (1992). Creative Cognition: Theory, Research, and Applications. Cambridge, MA: MIT Press

[B23] Fisher J. E. Mohanty A. Herrington J. D. Koven N. S. Miller G. A. Heller W. (2004). Neuropsychological evidence for dimensional schizotypy: implications for creativity and psychopathology. J. Res. Pers. 38, 24–31. 10.1016/j.jrp.2003.09.014

[B24] Heckers S. Barch D. M. Bustillo J. Gaebel W. Gur R. Malaspina D.. (2013). Structure of the psychotic disorders classification in DSM-5. Schizophr. Res. 150, 11–14. 10.1016/j.schres.2013.04.039 23707641

[B25] Heston L. L. (1966). Psychiatric disorders in foster home reared children of schizophrenic mothers. Br. J. Psychiatry 112, 819–825. 10.1192/bjp.112.489.819 5966555

[B26] Jauk E. Benedek M. Dunst B. Neubauer A. C. (2013). The relationship between intelligence and creativity: new support for the threshold hypothesis by means of empirical breakpoint detection. Intelligence 41, 212–221. 10.1016/j.intell.2013.03.003 23825884 PMC3682183

[B27] Jauk E. Benedek M. Neubauer A. C. (2014). The road to creative achievement: a latent variable model of ability and personality predictors. Eur. J. Pers. 28, 95–105. 10.1002/per.1941 24532953 PMC3923982

[B28] Jung R. E. Grazioplene R. Caprihan A. Chavez R. S. Haier R. J. (2010). White matter integrity, creativity, and psychopathology: disentangling constructs with diffusion tensor imaging. PLoS ONE 5:e9818. 10.1371/journal.pone.0009818 20339554 PMC2842439

[B29] Karlsson J. L. (1970). Genetic association of giftedness and creativity with schizophrenia. Hereditas 66, 177–182. 10.1111/j.1601-5223.1970.tb02343.x 5525825

[B30] Kaufman S. B. Paul E. S. (2014). Creativity and schizophrenia spectrum disorders. Front. Psychol. 5:1145. 10.3389/fpsyg.2014.01145 PMC4217346 25404921

[B31] Keefe J. A. Magaro P. A. (1980). Creativity and schizophrenia: an equivalence of cognitive processing. J. Abnorm. Psychol. 89, 390–398. 10.1037/0021-843X.89.3.390 7410706

[B32] Kim K. H. (2005). Can only intelligent people be creative? A meta-analysis. J. Second. Gift. Educ. 16, 57–66. 10.4219/jsge-2005-473

[B33] Kris E. (1952). Psychoanalytic Explorations in Art. New York, NY: International Universities Press

[B34] Kyaga S. Landén M. Boman M. Hultman C. M. Långström N. Lichtenstein P. (2013). Mental illness, suicide and creativity: 40-year prospective total population study. J. Psychiatr. Res. 47, 83–90. 10.1016/j.jpsychires.2012.09.010 23063328

[B35] Kyaga S. Lichtenstein P. Boman M. Hultman C. Långström N. Landén M. (2011). Creativity and mental disorder: family study of 300,000 people with severe mental disorder. Br. J. Psychiatry 199, 373–379. 10.1192/bjp.bp.110.085316 21653945

[B36] Merten T. Fischer I. (1999). Creativity, personality and word association responses: associative behaviour in forty supposedly creative persons. Pers. Individ. Dif. 27, 933–942. 10.1016/S0191-8869(99)00042-2

[B37] Nelson B. Rawlings D. (2010). Relating schizotypy and personality to the phenomenology of creativity. Schizophr. Res. 36, 388–399. 10.1093/schbul/sbn098 18682376 PMC2833116

[B38] Nettle D. (2006). Schizotypy and mental health amongst poets, visual artists, and mathematicians. J. Res. Pers. 40, 876–890. 10.1016/j.jrp.2005.09.004

[B39] Nusbaum E. C. Silvia P. J. (2011). Are intelligence and creativity really so different? Fluid intelligence, executive processes, and strategy use in divergent thinking. Intelligence 39, 36–45. 10.1016/j.intell.2010.11.002

[B40] Runco M. A. Jaeger G. J. (2012). The standard definition of creativity. Creat. Res. J. 24, 92–96. 10.1080/10400419.2012.650092

[B41] Schlesinger J. (2009). Creative mythconceptions: a closer look at the evidence for the “mad genius” hypothesis. Psychol. Aesthet. Crea. Arts 3, 62–72. 10.1037/a0013975

[B42] Silvia P. J. Winterstein B. P. Willse J. T. Barona C. M. Cram J. T. Hess K. I.. (2008). Assessing creativity with divergent thinking tasks: exploring the reliability and validity of new subjective scoring methods. Psychol. Aesthet. Crea. Arts 2, 68–85. 10.1037/1931-3896.2.2.68

[B43] Simonton D. K. (2000). Creativity: cognitive, personal, developmental, and social aspects. Am. Psychol. 55, 151–158. 10.1037/0003-066X.55.1.151 11392859

[B44] Simonton D. K. (2014). More method in the mad-genius controversy: a historiometric study of 204 historic creators. Psychol. Aesthet. Crea. Arts 8, 53–61. 10.1037/a0035367

[B45] Sussmann J. E. Lymer G. K. S. McKirdy J. Moorhead T. W. J. Maniega S. M. Job D.. (2009). White matter abnormalities in bipolar disorder and schizophrenia detected using diffusion tensor magnetic resonance imaging. Bipolar Disord. 11, 11–18. 10.1111/j.1399-5618.2008.00646.x 19133962

[B46] Weisberg R. W. (1999). Creativity and knowledge: a challenge to theories, in Handbook of Creativity, ed Sternberg R. J. (Cambridge, UK: Cambridge University Press), 226–250

